# Efficient context-dependent model building based on clustering posterior distributions for non-coding sequences

**DOI:** 10.1186/1471-2148-9-87

**Published:** 2009-04-30

**Authors:** Guy Baele, Yves Van de Peer, Stijn Vansteelandt

**Affiliations:** 1 Department of Applied Mathematics and Computer Science, Ghent University, Krijgslaan 281 S9, B-9000, Ghent, Belgium; 2 Department of Plant Systems Biology, VIB, B-9052, Ghent, Belgium; 3 Bioinformatics and Evolutionary Genomics, Department of Molecular Genetics, Ghent University, B-9052, Ghent, Belgium

## Abstract

**Background:**

Many recent studies that relax the assumption of independent evolution of sites have done so at the expense of a drastic increase in the number of substitution parameters. While additional parameters cannot be avoided to model context-dependent evolution, a large increase in model dimensionality is only justified when accompanied with careful model-building strategies that guard against overfitting. An increased dimensionality leads to increases in numerical computations of the models, increased convergence times in Bayesian Markov chain Monte Carlo algorithms and even more tedious Bayes Factor calculations.

**Results:**

We have developed two model-search algorithms which reduce the number of Bayes Factor calculations by clustering posterior densities to decide on the equality of substitution behavior in different contexts. The selected model's fit is evaluated using a Bayes Factor, which we calculate via model-switch thermodynamic integration. To reduce computation time and to increase the precision of this integration, we propose to split the calculations over different computers and to appropriately calibrate the individual runs. Using the proposed strategies, we find, in a dataset of primate Ancestral Repeats, that careful modeling of context-dependent evolution may increase model fit considerably and that the combination of a context-dependent model with the assumption of varying rates across sites offers even larger improvements in terms of model fit. Using a smaller nuclear SSU rRNA dataset, we show that context-dependence may only become detectable upon applying model-building strategies.

**Conclusion:**

While context-dependent evolutionary models can increase the model fit over traditional independent evolutionary models, such complex models will often contain too many parameters. Justification for the added parameters is thus required so that only those parameters that model evolutionary processes previously unaccounted for are added to the evolutionary model. To obtain an optimal balance between the number of parameters in a context-dependent model and the performance in terms of model fit, we have designed two parameter-reduction strategies and we have shown that model fit can be greatly improved by reducing the number of parameters in a context-dependent evolutionary model.

## Background

The past decades have seen the rise of increasingly complex models to describe evolution, both in coding and in non-coding datasets, using a range of different inferential methods of varying complexity. More accurate mathematical models of molecular sequence evolution continue to be developed for good reasons. First, the additional complexity of such models can lead to the identification of important evolutionary processes that would be missed with simpler models. Such discoveries may increase our understanding of molecular evolution. Second, using more accurate models may help to infer biological factors, such as phylogenetic topologies and branch lengths, more reliably. This may arise from the improved ability of those complex models to account for factors that simpler models neglect and whose influence on observed data might otherwise be misinterpreted [1].

Currently, the importance of modeling varying rates across sites in recovering the correct tree topology is well-known (see e.g. [2]). Acknowledging that the evolutionary rate at different sites might differ may, however, not be sufficient. Takezaki and Gojobori [3] used concatenated sequences of all protein-coding genes in mitochondria to recover the phylogeny of 28 vertebrate species. When the tree was rooted by lampreys or lampreys and sea urchins, the root of the vertebrate tree was incorrectly placed in the maximum-likelihood tree even when accounting for varying rates across sites. The authors suggest the importance of using the appropriate model for probabilities of substitution among different amino acids or nucleotides, as well as the assumption of varying rates across sites. Several other studies confirm the importance of using appropriate evolutionary models (see e.g. [4,5]).

In this article, we focus specifically on relaxing the assumption of site-independent evolution, motivated by the fact that a number of empirical studies have found this assumption to be overly restrictive (e.g. [6-11]). Also the detection of the CpG-methylation-deamination process in mammalian data has given rise to many context-dependent studies (for an overview of such so-called CpG-related studies, studies using codon-based models, as well as the empirical studies mentioned, see [12]). In our previous work [12], we have introduced a context-dependent approach using data augmentation which builds upon standard evolutionary models, but incorporates site dependencies across the entire tree by letting the evolutionary parameters in these models depend upon the ancestral states at the two immediate flanking sites. Indeed, once that ancestral sequences have been estimated, the evolution of a given site across a branch is allowed to depend upon the identities of its immediate flanking bases at the start (i.e. the ancestor) of that branch. The use of existing evolutionary models avoids the need for introducing new and high-dimensional evolutionary models for site-dependent evolution, such as those proposed by Siepel and Haussler [13] and Hwang and Green [14]. Indeed, using a general time-reversible (GTR) model, which contains six evolutionary parameters on top of the four base frequencies used in the model, for each of the 16 neighboring base compositions results in a total of 96 parameters (although the GTR model is often regarded to have five free parameters, which results in 80 parameters instead of 96). This number of parameters does not include the set of four stationary equilibrium frequencies, which is assumed to be context-independent. Using a Markov chain Monte Carlo approach with data augmentation, one may then infer the evolutionary parameters under the resulting model for a large genomic dataset under a fixed tree topology. Previous analyses [12] based on this model have revealed large variations in substitution behavior dependent upon the neighbouring base composition.

The increase in dimensionality of such context-dependent models warrants model reduction strategies [15] based on merging similar evolutionary contexts. One approach is to evaluate Bayes Factors [16] to compare models with and without merged contexts. Here, the Bayes Factor is a ratio of two marginal likelihoods (i.e. two normalizing constants of the form *p*(*Y*_*obs*_|*M*), with *Y*_*obs *_the observed data and *M *an evolutionary model under evaluation) obtained under the two models, *M*_0 _and *M*_1_, to be compared [16,17]:



Bayes Factors greater (smaller) than 1 suggest evidence in favor of *M*_1 _(*M*_0_). In this paper, we will use log Bayes Factors, which are typically divided into 4 categories depending on their value: from 0 to 1, indicating nothing worth reporting; from 1 to 3, indicating positive evidence of one model over the other; from 3 to 5, indicating strong evidence of one model over the other; and larger than 5, indicating significant (or very strong) evidence of one model over the other [16].

We have chosen to calculate Bayes Factors using thermodynamic integration [18], since the harmonic mean estimator of the marginal likelihood systematically favors parameter-rich models. Thermodynamic integration is a generalization of the bridge sampling approach and is therefore often referred to as 'path-sampling' (see e.g. [19-21]). Lartillot and Phillipe [18] present two methods to calculate the Bayes Factor between two models. Using their so-called annealing or melting approach one model at a time is evaluated, resulting in a marginal likelihood for each model. The ratio of these individual marginal likelihoods then yields a Bayes Factor. When this approach yields marginal likelihoods with large error estimates, the resulting log Bayes Factor can be inaccurate. This can be avoided by using model-switch thermodynamic integration, which directly calculates the log Bayes Factor (Lartillot and Philippe, 2006). By construction, this approach results in lower error estimates for the Bayes Factor and allows one to make use of the additivity property of the logarithmic function to calculate Bayes Factors [22].

Unfortunately, building models based on Bayes Factor-based model comparisons is not feasible because calculating Bayes Factors requires vast amounts of computation time. In view of this, we reduce the number of model evaluations by proposing two schemes for clustering posterior density estimates from different evolutionary contexts (and thus for merging these contexts). To evaluate the fit of the resulting model, a Bayes Factor must be calculated. We propose 2 adaptations of the thermodynamic integration method proposed by Lartillot and Philippe [18] to make this practically feasible. First, we show how the Bayes Factor calculation can be performed in parallel independent runs on different nodes in a cluster system, thus greatly reducing the time needed to obtain results. Second, we show that these independent runs can be adjusted depending on the part of the integrand that is being integrated to allow for more intensive calculations in hard-to-evaluate parts of the integrand in the model-switch integration procedure, resulting in more accurate (log) Bayes Factor estimates.

## Methods

### Data

We analyze two datasets which we have discussed in earlier work [12]. The first dataset consists of 10 sequences from vertebrate species, each consisting of 114,726 sites, and is analyzed using the following rooted tree topology (((((Human, Chimpanzee), Gorilla), Orang-utan), ((Baboon, Macaque), Vervet)), ((Marmoset, Dusky Titi), Squirrel Monkey)). We refer to this dataset as the 'Ancestral Repeats' dataset. The second dataset consists of 20 small subunit (SSU) rRNA genes (nuclear), consists of 1,619 sites for each sequence and is analyzed using the 50% majority rule posterior consensus tree obtained under the general time-reversible model. This dataset contains the following sequences: *Cyanophora paradoxa, Nephroselmis olivacea, Chlamydomonas moewusii, Volvox carteri, Paulschulzia pseudovolvox, Coleochaete orbicularis 2651, Coleochaete solute 32d1, Coleochaete irregularis 3d2, Coleochaete sieminskiana 10d1, Zygnema peliosporum, Mougeotia sp 758, Gonatozygon monotaenium 1253, Onychonema sp 832, Cosmocladium perissum 2447, Lychnothamnus barbatus 159, Nitellopsis obtusa F131B, Chara connivens F140, Lamprothamnium macropogon X695, Arabidopsis thaliana and Taxus mairei*. We refer to this dataset as the 'Nuclear SSU rRNA' dataset.

### Evolutionary models

We have used the general time-reversible model (GTR; [23]) to study site interdependencies, with the following substitution probabilities:



with *π *= {*π*_*A*_, *π*_*C*_, *π*_*G*_, *π*_*T*_} the set of base frequencies and *rAC*, *rAG*, *rAT*, *rCG*, *rCT *and *rGT *the evolutionary substitution parameters. As in our previous work (Baele et al., 2008), let *θ *= {2*π*_*A *_*π*_*G *_*rAG*, 2*π*_*A *_*π *_*C *_*rAC*, 2*π*_*A *_*π*_*T *_*rAT*, 2*π*_*G *_*π*_*C *_*rCG*, 2*π*_*G *_*π*_*T *_*rGT*, 2*π*_*C *_*π*_*T *_*rCT*} be the terms of the scaling formula that binds the parameters of the model and *T *be the set of branch lengths with *t*_*b *_(*t*_*b *_≥ 0) one arbitrary branch length and *μ *a hyperparameter in the prior for *t*_*b *_in *T*. As in Baele et al. (2008), the following prior distributions *q*(·) were chosen for our analysis, with Γ(.) the Gamma function:

*π *~ Dirichlet (1,1,1,1), *q *(*π*) = Γ (4) on ,

*θ *~ Dirichlet (1,1,1,1,1,1), *q *(*θ*) = Γ (6) on ,

*t*_*b*_|*μ *~ Exponential (*μ*),  for each *t*_*b *_in *T*

and

*μ *~ Inv-gamma (2.1, 1.1), , *μ *> 0.

Branch lengths are assumed i.i.d. given *μ*. When the model allows for the presence of multiple contexts of evolution, each context is assumed to have its own prior, independently of other contexts.

As there are 16 possible neighboring base combinations, we use a distinct GTR model per neighboring base composition, thus increasing the number of evolutionary contexts from 1 to16 for a full context-dependent model (Baele et al., 2008). The goal of this article is to reduce the dimension of such a model in an accurate and computationally efficient manner, by sharing parameters between contexts, which will improve the fit to the data. Note that the independent GTR model will be used as the reference model (i.e. the model to which all other models will be compared) throughout the remainder of this paper.

### Thermodynamic integration – split calculation

The split-calculation approach discussed in this section can be skipped by the less technically-minded people.

Different context-dependent models can be compared in terms of model fit with the independent GTR model by calculating the appropriate (log) Bayes Factors. One may use model-switch thermodynamic integration for this purpose [18]. This is a computationally intensive approach which yields reliable estimates for the (log) ratio of the marginal likelihoods corresponding to two models. Below, we explain how one can use a split calculation approach to make this integration procedure computationally more tractable.

Suppose our goal is to calculate the Bayes Factor corresponding to models *M*_0 _and *M*_1 _defined on the same parameter space Θ. The true data densities (conditional on the parameter *θ*) are denoted by

(1)

for the models *M*_*i*_, i = 0, 1, where *q*_*i*_(*θ*) denotes the joint density of the observed data and the parameter *θ*, and

(2)

is a normalizing constant. The latter encodes the marginal data density, which is needed in the calculation of the (log) Bayes Factor. The key idea behind model-switch integration is to translate the problem of integration w.r.t. *θ *into the relatively simpler problem of averaging over *θ*. For this purpose, a continuous and differentiable path (*q*_*β *_(*θ*))_0≤*β*≤1 _(with corresponding *p*_*β *_(*θ*) and *Z*_*β *_(*θ*)) is chosen in the space of unnormalized densities, joining *q*_0_(*θ*) and *q*_1_(*θ*), which thus goes directly from model *M*_0 _to model *M*_1_. When *β *tends to 0 (resp. 1), *p*_*β *_(*θ*) converges pointwise to *p*_0 _(*θ*) (resp. *p*_1 _(*θ*)), and *Z*_*β *_(*θ*) to *Z*_0_(*θ*) (resp. *Z*_1_(*θ*)). The log Bayes Factor of model *M*_1 _versus *M*_0 _can now be calculated as the log-ratio [18]

(3)

where *E*_*β *_[...] denotes the expectation with respect to *θ *under the density *p*_*β *_(*θ*), and with *U*(*θ*) the potential

(4)

This expectation *E*_*β *_[*U*(*θ*)]may be approximated with a sample average once a sample of random draws from *p*_*β *_(*θ*) is obtained using MCMC. The integration problem is now simplified to the problem of integrating w.r.t. a scalar parameter *β*, which is relatively easily handled by numerical approximation using the composite trapezoidal rule.

These calculations can be partitioned over a number of computers upon rewriting the integral in expression (3) as

(5)

with *α*_0 _= 0 <*α*_1_, < ... <*α*_*n *_< 1 = *α*_*n*+1 _dividing the interval [0,1] into *n *subintervals with the number of MCMC-updates of *β *in each subinterval resp. equal to chosen values *K*_0_, *K*_1_,..., *K*_*n*_. For each value of *β*, the Markov chain is updated during a number of *Q *iterations (during which *β *is held constant), after which *β *is increased (or decreased). As in the work of Lartillot and Philippe [18], each of these integrals can be calculated using the composite trapezoidal rule to obtain the so-called quasistatic estimator

(6)

for the m^th ^subinterval, with *δ*_*m = *_*α*_*m*+1 _-*α*_*m*_, (*θ*_*i*_), *i *= *p*...*r *(with ) the saved parameter draws. A possible approach to calculate the quasistatic estimator is thus to save the set of parameter values in the iteration before *β *is increased (or decreased), this way obtaining a set of parameter values *θ*_*i *_for each value of *β *during the transition of *β *from *α*_*m *_to *α*_*m*+1_. Calculating this quasistatic estimator for each subintegral and adding yields the following expression for the quasistatic estimator of :

(7)

The obtained estimates of the log Bayes Factor are subject to a discretization error (due to numerical integration) and sampling variance (due to the limited number of MCMC-draws used in the calculation of the expected potential). Below we report on how to quantify these errors under the split calculation algorithm proposed above. The discretization error of  is characterized by its worst-case upper (resp. lower) error which, because *E*_*β *_[*U*(*θ*)] is monotone in *β*, is given by the area between the piecewise continuous function joining the measured values of *E*_*β *_[*U*(*θ*)] and the upper (resp. lower) step function built from them [18]. Both areas (i.e. between both upper and lower step functions and the continuous function) are equal to:

(8)

By splitting the calculation over different subintervals, we obtain a sum of discretization errors, one for each integral, which is given by

(9)

The sampling variance can be estimated by summing the variances over the parallel chains

(10)

assuming independence between the successive draws from the chain. The total error on the log Bayes Factor equals *σ *= *σ*_*d *_+ 1.645 *σ*_*s*_, with *σ*_*s *_the square root of the sampling variance [18]. In general, a sufficiently long burn-in is necessary to obtain reliable estimates and low error margins.

### Data augmentation

Because of the computational complexity, Baele et al. [12] use data augmentation for estimating the parameters of a context-dependent model, whereby ancestral data are repeatedly imputed. Indeed, the computational complexity of using context-dependent models does not allow for easy calculation of the observed data likelihood and requires the use of a full (or complete) data likelihood to make inference possible. As a result, each missing ancestor in the tree needs to be provided with an estimated ancestral nucleotide in each iteration. This has implications for the model-switch thermodynamic integration scheme, which was developed for settings where inference is based on the observed data likelihood [18]. In our approach, i.e. data augmentation with model-switch thermodynamic integration, the ancestral data can be shared between both models (i.e. the imputations take identical values under both models) and in that case must be part of the unknown parameter *θ*. In particular, each ancestral "augmented" site is imputed with a draw from a multinomial distribution from the probability density *p*_*β *_(*θ*) since the expectation *E*_*β *_[*U*(*θ*)] will be approximated with a sample average of random draws from *p*_*β *_(*θ*) [18]. In our approach, this probability density for the ancestral site *i *has the following form (with *Y*_*mis*, *i *_representing the state of the ancestor that is being augmented at site *i*, Y_*mis*,-*i *_representing the set of states for all remaining ancestors, *r*_*i *_the evolutionary rate at site *i *and *β *the current position along the path between the two posterior distributions)

(11)

Upon noting that *p*_*β *_(*θ*) = *q*_*β *_(*θ*)/∫*q*_*β *_(*θ*), expression (11) yields

(12)

In our approach, we choose *q*_*β *_(*Y*_*mis*_, {*r*_*i*_}, *Y*_*obs*_|*M*_0_, *M*_1_) = (*L*_*X*_|*M*_0_)^1-*β*^(*L*_*X*_|*M*_1_)^*β*^, implying that each ancestral "augmented" site is imputed with a draw from a multinomial distribution with probability

(13)

where *L*_*X*_|*M*_*i *_is the complete data likelihood under model *M*_*i *_when *X *∈ {*A*, *C*, *G*, *T*} is the value augmented for the considered ancestral site. This result in a probability for each nucleotide to be inferred at a given site, with the four probabilities summing to one. The ancestral sequences are then updated sequentially, i.e. one site at a time, from top to bottom in the considered tree and from the first site moving along the sequence up to the last site, each ancestral site is updated during each update cycle.

When *β *equals 0 (1), the ancestral sequences are random draws from the posterior distribution of *Y*_*mis *_under *M*_0 _(*M*_1_). At certain ancestral positions, this may result in imputed values with small likelihoods under *M*_1 _(*M*_0_), which in turn leads to larger differences between the log likelihoods of the two models. Because of this, the contributions of the model-switch integration scheme to the log Bayes Factor are most tedious to calculate when *β *is close to 0 and 1, which is why we use smaller update steps for *β *in those situations. In the case of an observed data likelihood, which involves summing over all missing ancestral nucleotides, this situation does not occur.

### Evolutionary rate augmentation

To accommodate varying rates across sites (or among-site rate variation), we use a similar data augmentation approach as before, which now additionally imputes evolutionary rates in order to avoid summing the likelihood over all the possible rate classes. Given a discrete approximation to the gamma distribution with *n *rate classes (where *n *= 1 encodes the assumption of equal rates), the rate *r*_*i *_at each site *i *for model *M*_1 _is updated by drawing from a multinomial distribution with probability

(14)

where *r*_*i *_represents the rate of site *i*, which is being augmented, *r*_-*i *_represents the set of rates for all remaining sites, *β *represents the current position along the path between the two posterior distributions, and  is the complete data likelihood under the rates-across-sites model *M*_1 _when *X *∈ {*r*_1_,..., *r*_*n*_} is the value imputed for the considered missing rate. Note that, when comparing model *M*_1 _with a model which assumes equal rates, only the rate parameters indexing *M*_1 _need to be updated with a new set of rates at each model-switch iteration in the calculation of a Bayes Factor.

### Context reduction

Our context-dependent model consists of 16 possibly different GTR models, one for each neighbouring base composition (a.k.a. 'evolutionary context'). In practice, it is likely that the evolutionary processes are similar in a number of neighboring base compositions, or that the data are insufficiently informative to distinguish these. This suggests reducing the model's dimensionality by merging contexts, which may subsequently lead to evolutionary models with reduced parameter uncertainty which fit the data better than the independent model. Unfortunately, the time-consuming calculation of Bayes Factors makes exhaustive model search using Bayes Factors currently prohibiting. In view of this, we have sampled 1,000 values of each of the 96 parameters in our full context-dependent model from the Markov chain every 50^th ^iteration after an initial burn-in of 50,000 iterations. On the basis of the 1,000 values for each of the six parameters per context, the first two principal components are calculated and displayed in a scatterplot, thus resulting in 16 six-dimensional clusters each consisting of one context.

The location of certain contexts in such a scatterplot may indicate strong differences between some contexts, but not between others, and may thus be informative of contexts that can meaningfully be merged. However, this is not always the case partly because information is inevitably lost by considering only two principal components. Using a scatterplot matrix of the first three principal components might add information, but would still require arbitrary decisions from the researcher on the clustering of different contexts. In this section, we therefore propose two algorithmic, automated methods for clustering contexts by progressive agglomeration. Each decision taken by these algorithms is then confirmed by calculating the corresponding log Bayes Factor.

### A likelihood-based reduction approach

The parameters in each of the 16 neighboring base compositions can be described by a six-dimensional mean with corresponding variance-covariance matrix. Assuming a multivariate normal posterior density within each context (which is asymptotically valid), a likelihood function of all sampled parameter values can thus be calculated. This initial likelihood is the starting point for our first reduction approach, which uses the following iteration scheme:

1. Reduce the number of contexts with 1 by merging 2 contexts. Calculate the likelihood of the corresponding model. Repeat this for all pairs of contexts.

2. Select the highest likelihood obtained in the previous step, merge the two corresponding clusters and recalculate the empirical mean and variance-covariance matrix of the parameters corresponding to the merged clusters. To make the calculations more feasible, we do not enforce to run a Markov chain for each newly obtained model to infer new estimates of the posterior means and variance-covariance matrices.

3. Iterate steps 1 and 2 until only one cluster remains.

Through the remainder of this work, we define a cluster as the merge of two or more evolutionary contexts. As the merging of clusters progresses, the likelihood will gradually decrease in value. This is expected as the parameter estimates can be better approximated by context-specific means and variance-covariance matrices instead of cluster-specific means and variance-covariance matrices. Since the likelihood only decreases (and additionally depends on the chosen number of samples in an arbitrary fashion), it cannot be used to determine the optimal number of clusters/contexts. In terms of the (log) Bayes Factor, it is typically expected that the model fit will first gradually increase, reach an optimum, and then decrease. In each step of the algorithm, we therefore calculate the Bayes Factor corresponding to the selected model. In principle, the algorithm can be stopped when the Bayes Factors decrease convincingly with additional context reductions.

In each step of the above algorithm, the number of parameters in the model decreases with 6. Since each step selects the clustering which minimizes the decrease in log likelihood, this approach is likely to detect a model with near-optimal (log) Bayes Factor.

### A graph-based reduction approach

While the parameter-reduction approach of the previous section has a statistical basis, it is likely to yield models with suboptimal fit. Indeed, the likelihood-based approach systematically favors merging two separate contexts over merging a context with already merged contexts (to build clusters with three or more contexts). Consider a scenario of two proposed clusters, one already containing two contexts and being expanded with a third context, and one containing a single context and being merged with another context. These two clusters will each have one mean and one variance-covariance matrix to represent the parameter estimates. However, the three-context cluster is not represented so easily with this reduced parameterization due to the increased variance of the three-contexts cluster. Such an increase in variance leads to a drastic decrease of the likelihood which implies that merging small clusters will tend to be preferred over expanding existing clusters.

This artifact may be resolved by re-running a Markov chain after each merge using the newly obtained model, which will allow to re-estimate the posterior variance-covariance matrices needed to predict the next merge operation. However, this requires additional computational efforts, making it much less suited for reducing the model complexity. We thus propose the following graph-based approach, which avoids the need for re-estimating the posterior variance of each cluster by using the loglikelihood difference between the 16-context model and each 15-context model as costs in a graph-based algorithm. It requires one Markov chain run to predict all necessary clustering steps, to determine a possibly more optimal solution:

1. Calculate the likelihoods of all possible pair wise context reductions (120 in total), starting from the initial clustering of each context separately (which yielded the initial likelihood).

2. Use the difference between the newly obtained likelihoods and the initial likelihood as edge costs in a fully connected undirected graph of 16 nodes. Similar contexts will be connected by low-cost edges while dissimilar contexts will be connected by high-cost edges. The cost function then consists of the sum of each edge that participates in a cluster (i.e. one edge for a cluster of two contexts, three edges for a cluster of three contexts, six edges for a cluster of four contexts ...).

3. Sort the 120 likelihood differences, smallest differences first (each difference corresponds with a merge of two clusters).

4. Using the sorted list, merge the clusters following the order proposed by the list. If the proposed merge is between two contexts which both have not yet participated in a merge operation, proceed with merging them and color the two nodes and the connecting edge in the graph. If at least one of the contexts is already colored in the graph, proceed with the merging of the clusters if the resulting fully interconnected network in the graph (i.e. between the contexts to be merged) yields the lowest value for the cost function of the possible networks of similar structure (i.e. the fully connected networks with an equal amount of nodes) that have not yet been colored (see the Results section for a practical example). If there is a lower-cost alternative, do not merge the clusters and proceed with the following entry from the list, until only one cluster remains. The objective of this graph-based approach is thus not simply to minimize the cost function, but to minimize the cost function conditional on the proposed new clustering. This means that when a proposed merge is accepted, it was the cheapest merge possible between competing networks of similar structure.

This graph-based reduction approach has the advantage that it does not need additional Markov chains to be run. Given the costs of the various reductions and their order to be evaluated in, determined in the first two steps, the algorithm attempts to cluster those contexts closest to one another, with the enforced constraints that only competing clusters of the same composition are compared. This should result in larger clusters and thus possibly in larger improvements of the model fit.

## Results and discussion

### The Ancestral Repeats dataset

#### Approaches Compared

**Figure 1:**
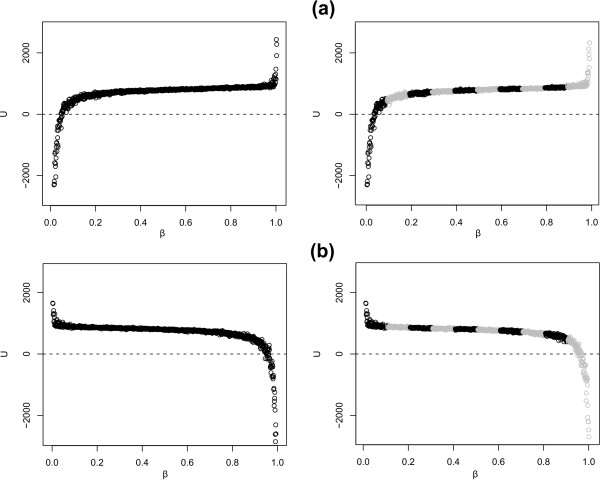
**Model-switch integration for the Ancestral Repeats dataset**. Results for the two model-switch integration schemes: (a) annealing, i.e. *β *increases from 0 (independent GTR model) to 1 (GTR16C full context-dependent model) and (b) melting, i.e. *β *decreases from 1 to 0. The comparison between a single run (left) and a composite run using ten intervals (right) reveals almost identical log Bayes Factor estimates. The composite run yields a slightly broader confidence interval around the log Bayes Factor estimate.

To compare our Bayes Factor calculation approach with the original approach of Lartillot and Philippe [18], we have initially opted for a constant increment for *β *and an equal number of *Q *updates for all the parameters and ancestral sites to estimate the log Bayes Factor and its error for the large Ancestral Repeats dataset. The results are shown in Table 1 and in Figure 1. Thermodynamic integration requires a drastic increase in CPU time compared to a plain posterior sampling under the more demanding of the two models that are being compared [18]. This requires running a chain for several days, up to several weeks for more complex models (where mixing can be more challenging). A single-run log Bayes Factor calculation with low accuracy (i.e. only *Q *= 200 updates for each value of *β*, with step size 0.001) of the full 16-contexts model (GTR16C) against the independent GTR model takes 42 days for one direction (i.e. either annealing or melting) on a single computer, given the large number of sites. More accurate settings for the model-switch integration will further increase calculation time and are in fact necessary as the log Bayes Factor estimates in both directions, i.e. 592.7 and 693.6, are still far apart. In contrast, our proposed approach yields very similar results in terms of the log Bayes Factor estimates in both directions as can be seen in Table 1. The calculation time for our approach is reduced to 6 days on 10 cluster nodes and further reductions are possible since we opted for a lengthy burn-in sequence of 10,000 iterations. Its only disadvantage lies in slightly broader confidence intervals for the log Bayes Factor, which is an expected consequence of using several independent chains.

**Table 1:** Comparison of single versus composite run Bayes Factor estimation.

Annealing Integration					
*δβ *= 0.001	Integration interval	Q.E.	σ_d_	σ_s_	total error

Q = 200	0.00 – 0.10	-121.7	11.7	2.0	14.9
	0.10 – 0.20	57.3	0.1	0.4	0.7
	0.20 – 0.30	68.9	0.0	0.2	0.4
	0.30 – 0.40	74.3	0.0	0.2	0.3
	0.40 – 0.50	77.8	0.0	0.2	0.3
	0.50 – 0.60	80.6	0.0	0.1	0.2
	0.60 – 0.70	82.1	0.0	0.1	0.2
	0.70 – 0.80	83.9	0.0	0.1	0.2
	0.80 – 0.90	87.0	0.0	0.2	0.3
	0.90 – 1.00	102.7	1.3	1.5	3.8

Total	0.00 – 1.00	592.9	13.2	4.9	21.3

Composite Run log Bayes Factor: 592.9					
**Composite Run Confidence Interval: [571.6; 614.2]**					

Single Run log Bayes Factor: 592.7. σ_d _= 13.4. σ_s _= 2.5. *σ *= 17.6					
**Single Run Confidence Interval: [575.1; 610.2]**					

Melting Integration					

*δβ *= 0.001	Integration interval	Q.E.	σ_d_	σ_s_	total error

Q = 200	1.00 – 0.90	121.7	8.2	3.3	13.7
	0.90 – 0.80	87.5	0.0	0.2	0.3
	0.80 – 0.70	84.5	0.0	0.1	0.2
	0.70 – 0.60	82.7	0.0	0.1	0.2
	0.60 – 0.50	80.8	0.0	0.1	0.2
	0.50 – 0.40	77.9	0.0	0.2	0.3
	0.40 – 0.30	74.5	0.0	0.2	0.3
	0.30 – 0.20	69.1	0.0	0.2	0.4
	0.20 – 0.10	58.1	0.1	0.4	0.7
	0.10 – 0.00	-42.9	3.2	1.4	5.6

Total	1.00 – 0.00	693.8	11.7	6.2	21.9

Composite Run log Bayes Factor: 693.8					
**Composite Run Confidence Interval: [671.9; 715.7]**					

Single Run log Bayes Factor: 693.6. σ_d _= 12.3. σ_s _= 3.6. *σ *= 18.3					

**Single Run Confidence Interval: [675.3; 711.9]**					

Comparison of single versus composite run Bayes Factor estimation reveals virtually identical log Bayes Factors, but tighter confidence intervals for the single run calculation, both in the annealing and melting scheme of the thermodynamic integration approach. A constant increment *δβ *(or decrement) of 0.001 was used for *β *with *Q *= 200 iterations for each value of *β*. Q.E. is the quasistatic estimator for each (thermodynamic) integration interval with discrete and sampling error denoted by σ_d _and σ_s_, respectively.

As can be seen from Table 1, running 200 full chain updates at each value of *β *works well only in the integration interval [0.1;0.9]. Indeed, the quasistatic estimates in both directions produce very similar results when *β *is in the interval [0.1;0.9]. However, as a result of the ancestral data augmentation, the same settings for *β *should not be applied when one of the models converges to its prior, i.e. in the integration intervals [0.0;0.1] and [0.9;1.0] for *β*, as the ancestral sites imputed in those intervals converge towards essentially arbitrary data for one of the models and yield very small values for the log likelihood. This makes it more difficult and time-consuming to yield similar and reliable estimates for both directions of the model-switch integration scheme. We have therefore opted to split our calculations into 20 sections, each with the same amount of chain updates (*Q *= 200), but using a larger number of updates for *β *as the chain progresses closer to one of the prior distributions. A referee remarked that Lepage et al. [24] have used a sigmoidal schedule for *β *to circumvent this problem.

The results for each of the subintervals and in each direction are reported in Tables 2 and 3. The log Bayes Factor estimates in both directions are now 630.7 (95% CI: [623.2; 638.2]) and 653.7 (95% CI: [645.5; 661.9]). Given the magnitude of the increase in terms of model fit, we have refrained from increasing the converging times in order to obtain more similar confidence intervals and we have taken the average of both estimates to be the resulting log Bayes Factor. Further, the width of the confidence intervals has decreased from 35 to 15 log units, suggesting that this approach also reduces the variance(s) of the log Bayes Factor estimates.

**Table 2:** Split calculation for the annealing model-switch integration.

*δβ*	Integration interval	Q.E.	σ_d_	σ_s_	total error
0.0001	0.00–0.01	-71.7	1.0	0.5	1.8
0.0001	0.01–0.02	-16.8	0.1	0.2	0.4
0.0002	0.02–0.04	-8.8	0.0	0.2	0.4
0.0002	0.04–0.06	0.8	0.0	0.2	0.3
0.0002	0.06–0.08	5.5	0.0	0.1	0.2
0.0002	0.08–0.10	7.9	0.0	0.1	0.2
0.001	0.10–0.20	57.3	0.1	0.4	0.7
0.001	0.20–0.30	68.9	0.0	0.2	0.4
0.001	0.30–0.40	74.3	0.0	0.2	0.3
0.001	0.40–0.50	77.8	0.0	0.2	0.3
0.001	0.50–0.60	80.6	0.0	0.1	0.2
0.001	0.60–0.70	82.1	0.0	0.1	0.2
0.001	0.70–0.80	83.9	0.0	0.1	0.2
0.001	0.80–0.90	87.0	0.0	0.2	0.3
0.0002	0.90–0.92	17.9	0.0	0.0	0.1
0.0002	0.92–0.94	18.0	0.0	0.0	0.1
0.0002	0.94–0.96	18.4	0.0	0.1	0.1
0.0002	0.96–0.98	19.1	0.0	0.1	0.2
0.0001	0.98–0.99	10.3	0.0	0.1	0.2
0.0001	0.99–1.00	18.1	0.3	0.5	1.1

Total	0.00–1.00	630.7	1.6	3.6	7.5

Composite Run log Bayes Factor: 630.7					
**Composite Run Confidence Interval: [623.2; 638.2]**					

Using a constant number of *Q *= 200 iterations per *β*, the contribution of each integration interval to the Bayes Factor value was calculated on a separate processor. This leads to an improved approximation of the contribution for the intervals [0.0; 0.1] and [0.9; 1.0] and also decreases the width of the confidence interval from 42.6 to 15.0 The increment *δβ *was allowed to change and *Q *= 200 iterations were performed for each value of *β*. Q.E. is the quasistatic estimator for each integration interval with discrete and sampling error denoted by *σ*_*d *_and σ_s_, respectively.

**Table 3:** Split calculation for the melting model-switch integration.

*δβ*	Integration interval	Q.E.	σ_d_	σ_s_	total error
0.0001	1.00–0.99	22.0	1.2	0.6	2.2
0.0001	0.99–0.98	10.2	0.0	0.1	0.2
0.0002	0.98–0.96	19.2	0.0	0.1	0.2
0.0002	0.96–0.94	18.5	0.0	0.1	0.1
0.0002	0.94–0.92	18.1	0.0	0.0	0.1
0.0002	0.92–0.90	17.8	0.0	0.0	0.1
0.001	0.90–0.80	87.5	0.0	0.2	0.3
0.001	0.80–0.70	84.5	0.0	0.1	0.2
0.001	0.70–0.60	82.7	0.0	0.1	0.2
0.001	0.60–0.50	80.8	0.0	0.1	0.2
0.001	0.50–0.40	77.9	0.0	0.2	0.3
0.001	0.40–0.30	74.5	0.0	0.2	0.3
0.001	0.30–0.20	69.1	0.0	0.2	0.4
0.001	0.20–0.10	58.1	0.1	0.4	0.7
0.0002	0.10–0.08	8.0	0.0	0.1	0.2
0.0002	0.08–0.06	5.6	0.0	0.1	0.2
0.0002	0.06–0.04	1.3	0.0	0.2	0.3
0.0002	0.04–0.02	-8.7	0.1	0.2	0.5
0.0001	0.02–0.01	-15.5	0.1	0.2	0.4
0.0001	0.01–0.00	-57.8	0.6	0.4	1.2

Total	0.00–1.00	653.7	2.3	3.6	8.2

Composite Run log Bayes Factor: 653.7					
**Composite Run Confidence Interval: [645.5; 661.9]**					

Using a constant number of *Q *= 200 iterations per *β*, the contribution of each integration interval to the Bayes Factor value was calculated on a separate processor. This leads to an improved approximation of the contribution for the intervals [1.0; 0.9] and [0.1; 0.0] and also decreases the width of the confidence interval from 43.9 to 16.3. The decrement *δβ *was allowed to change and *Q *= 200 iterations were performed for each value of *β*. Q.E. is the quasistatic estimator for each integration interval with discrete and sampling error denotes by *σ*_*d *_and σ_s_, respectively.

#### Varying rates across sites and CpG effects

To determine the impact of assuming varying rates across sites on the model fit, we calculated the log Bayes Factor comparing the independent GTR model with equal rates to the GTR model with varying rates across sites using *n *= 4 discrete rate classes, as proposed by Yang [25]. The log Bayes Factor equals 355.6, indicating a very strong preference towards the varying rates across sites assumption using four discrete rate classes. The mean estimate for the shape parameter of the gamma distribution using four rate classes equals 1.156 (Baele et al., 2008). Because four rates classes may not be sufficient to approximate the continuous gamma distribution, we have gradually increased the number of rate classes *n *as reported in Table 4. The log Bayes Factor equals 354.3 for *n *= 5, 354.6 for *n *= 6, 354.4 for *n *= 7 and 356.0 for *n *= 8 rate classes. Increasing the number of rate classes beyond *n *= 4 hence does not yield important improvements in model fit.

**Table 4:** Number of discrete rate classes when assuming varying rates across sites and the resulting increase in model fit obtained.

Model	Contexts	Annealing	Melting	log BF
GTR+G4	1 (7)	[334.8; 348.4]	[362.5; 376.7]	355.6
GTR+G5	1 (7)	[334.1; 348.1]	[360.4; 374.7]	354.3
GTR+G6	1 (7)	[333.6; 347.8]	[361.3; 375.7]	354.6
GTR+G7	1 (7)	[332.9; 347.1]	[361.6; 375.9]	354.4
GTR+G8	1 (7)	[334.0; 348.0]	[363.7; 378.3]	356.0
				
GTR	1 (6)	-	-	0

Starting from the default number of four rate classes for the discrete gamma approximation (GTR+G4), we have tested increasing numbers of rate classes. A minor improvement in model fit can be obtained by allowing for eight rate classes (GTR+G8), but such a small improvement may be due to discretization and sampling errors.

The previous results show that allowing for varying rates across sites drastically increases model fit compared to assuming equal rates for all sites. Analysis of the data using the context-dependent evolutionary model has further shown that substitution behavior is heavily dependent upon the neighbouring base composition [12]. A well-known context-dependent substitution process is the 5-methylcytosine deamination process (i.e., the CpG effect), which has been the subject of several studies (see e.g. [26-28]). We have calculated the log Bayes Factor of two different CpG effects. We have modeled the traditional CpG effect where the substitution behavior of a site can differ from the other sites when the 3' neighbor is guanine. The mean log Bayes Factor for this model, which contains a mere 12 parameters, equals 137.8 (annealing: [127.1; 141.0], melting: [137.0; 146.2]), a significant improvement in terms of model fit compared to the independent model. We have also modeled a CpG effect that is dependent upon its 5' neighbor, i.e. those sites with guanine as a 3' neighbor are assumed to have a different substitution behavior depending on the 5' neighbor. Such a model has 30 parameters and a mean log Bayes Factor of 157.8 when compared to the independent GTR model (annealing: [142.7; 155.0], melting: [159.6; 173.8]), i.e. this model is preferred over both the model assuming the traditional CpG effect and the independent model. However, the log Bayes Factor of 642.2 attained by the full context-dependent model (as compared to the independent GTR model) suggests that many more complex evolutionary patterns exist besides the CpG effect.

#### The likelihood-based reduction approach: results

**Figure 2:**
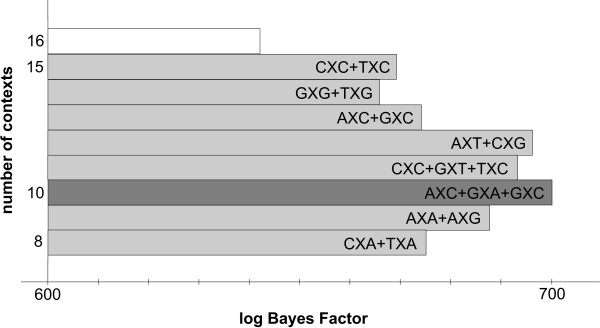
**Stepwise likelihood-based clustering of the Ancestral Repeats data**. The stepwise clustering of contexts using the likelihood-based clustering approach shows, from top to bottom, the subsequent merges of contexts for the Ancestral Repeats dataset. The starting point is a full (96-parameter) context-dependent model, shown in white. The optimal model has 10 clusters and 60 parameters.

**Figure 3:**
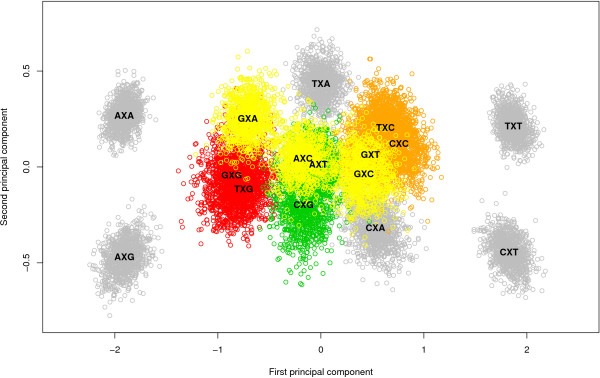
**Graphical representation of the likelihood-based optimal model for the Ancestral Repeats dataset**. The optimal model for the Ancestral Repeats dataset, as obtained by the likelihood-based clustering approach, has 10 clusters: 6 separate grey clusters (each containing a single context: AXA, AXG, CXA, CXT, TXA and TXT) and 4 colored clusters. The green cluster contains 2 contexts: AXT and CXG, the yellow cluster contains 3 contexts: AXC, GXC and GXA, the red cluster contains 2 contexts: GXG and TXG and the orange cluster contains 3 contexts: CXC, GXT and TXC.

Figure 2 shows the stepwise clustering of contexts with corresponding log Bayes Factors reported in Table 5. The full context-dependent model, consisting of 16 clusters each containing one context (denoted GTR16C), is shown by the white bar which corresponds to a log Bayes Factor of 642.2 (as compared to the independent GTR model). Each step of the algorithm yields a reduction of one context, resulting in the light grey bars in Figure 2, which are annotated with the new cluster structure that is being formed at that step. For example: in the first step, i.e. the reduction from 16 to 15 clusters (i.e. model GTR15C in Table 5), the CXC and TXC contexts are merged, reducing the number of parameters from 96 to 90. The log Bayes Factor of this 15-context model over the GTR model equals 669.2. In the second step, i.e. the reduction from 15 to 14 clusters (i.e. model GTR14C in Table 5), the GXG and TXG context are merged, further reducing the number of parameters to 84. While this 14-clusters model yields a lower log Bayes Factor over the GTR model (665.9) than the 15-clusters model, there is no reason to stop here as this decrease may well be the result of sampling and discretization error on the Bayes Factor and thus more optimal models might still be obtained by further context reduction. After further reductions, our likelihood-based reduction scenario yields an optimal clustering scheme for the Ancestral Repeats dataset consisting of 10 clusters (GTR10C; using a total of 60 parameters), as indicated by the dark grey bar in Figure 2. This 10-clusters-model yields a log Bayes Factor of 700.1 over the independent GTR model. The 10 clusters are shown in Figure 3, which identifies 6 separate single-context clusters (for the evolutionary contexts AXA, AXG, CXA, CXT, TXA and TXT) and 4 clusters consisting of two or three contexts. A first cluster contains 2 contexts: AXT and CXG, a second cluster contains 3 contexts: AXC, GXC and GXA, a third cluster contains 2 contexts: GXG and TXG, and a final cluster contains 3 contexts: CXC, GXT and TXC.

**Table 5:** Stepwise context reduction for the Ancestral Repeats dataset using the likelihood-based approach.

Model	Contexts	Annealing	Melting	log BF
GTR16C	16 (96)	[623.2; 638.2]	[645.5; 661.9]	642.2
				
GTR15C	15 (90)	[658.0; 672.1]	[665.0; 682.0]	669.2
GTR14C	14 (84)	[651.9; 668.4]	[664.3; 678.9]	665.9
GTR13C	13 (78)	[661.7; 675.9]	[672.6; 686.4]	674.2
GTR12C	12 (72)	[679.8; 694.7]	[695.8; 714.7]	696.3
GTR11C	11 (66)	[676.5; 692.7]	[694.4; 709.7]	693.3
**GTR10C**	**10 (60)**	**[686.0; 700.1]**	**[698.9; 715.5]**	**700.1**
GTR9C	9 (54)	[675.3; 689.0]	[685.5; 701.1]	687.7
GTR8C	8 (48)	[656.7; 669.7]	[678.5; 695.6]	675.1
				
GTR	1 (6)	-	-	0

The stepwise context reduction for the Ancestral Repeats dataset using the likelihood-based clustering approach reveals an optimal model with 10 clusters (GTR10C). It attains a log Bayes Factor of 700.1 (over GTR1C), which is a significant improvement over the full context-dependent model (GTR16C) that has 36 additional parameters. Further reducing the number of contexts decreases model fit.

#### The graph-based reduction approach: results

As predicted above, the likelihood-based reduction approach favors small clusters. To confirm this assumption, we have re-run a Markov chain using a context-dependent model consisting of the optimal number of 10 clusters derived using the likelihood-based approach. Using the parameter estimates from this model, we have calculated the posterior variances of the (yellow) cluster containing the AXC, GXA and GXC contexts and compared them to the empirical variances obtained from merging these three contexts but not re-running the chain. The actual posterior variances were much smaller, equaling merely between 3% and 24% of the empirical variances that were used. However, calculating these posterior variances is practically not feasible for fast model building because running a new Markov chain for the Ancestral Repeats dataset takes about 4 days per run. Further, the result of each run needs to be awaited to decide upon the next step in the clustering algorithm, which greatly increases the time needed to obtain an optimal context-dependent model.

**Figure 4:**
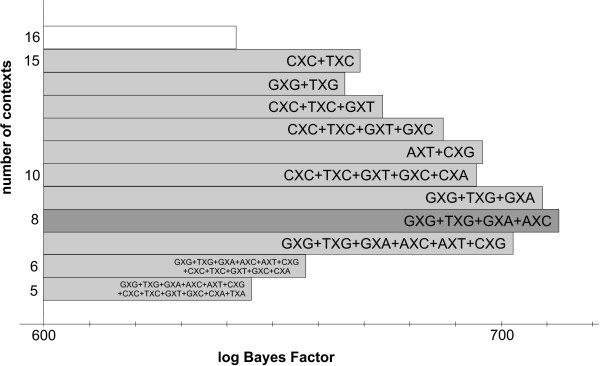
**Stepwise graph-based clustering of the Ancestral Repeats data**. The stepwise clustering of contexts using the graph-based clustering approach shows, from top to bottom, the subsequent merges of contexts for the Ancestral Repeats dataset. The starting point is a full (96-parameter) context-dependent model, shown in white. The optimal model has 8 clusters and 48 parameters.

In view of this, the graph-based reduction approach was designed. The decisions taken in the first 22 iterations are shown in Table 6, with corresponding log Bayes Factors in Figure 4 and Table 7. Starting from the full context-dependent model (GTR16C in Table 7), each step of the algorithm yields a reduction of one context, as shown by the light grey bars in Figure 4 which are annotated as in Figure 2. The first two reduction steps of the graph-based approach are identical to those of the likelihood-based approach. Further reductions show that fewer clusters are constructed by instead expanding existing clusters. The reduction to 13 clusters (GTR13C in Table 7), for example, consists of merging the previously separated GXT context with the cluster constructed in the first reduction step, thus creating a new cluster with 3 contexts: CXC, GXT and TXC.

**Table 6:** Determining the reduction path for the graph-based reduction approach.

Step	Context 1	Context 2	Log likelihood difference	Performed	Model
1	CXC	TXC	872.10	YES	GTR15C
2	GXG	TXG	1064.87	YES	GTR14C
3	GXT	TXC	1300.93	YES	GTR13C
4	CXC	GXC	1524.96	YES	GTR12C
5	AXC	GXC	1750.50	NO	
6	GXC	TXC	1777.24	SKIP	
7	AXT	CXG	1868.77	YES	GTR11C
8	CXC	GXT	1925.79	SKIP	
9	CXA	GXT	1928.98	YES	GTR10C
10	GXC	GXT	1940.97	SKIP	
11	GXA	TXG	1961.01	YES	GTR9C
12	AXC	GXA	1964.73	YES	GTR8C
13	GXA	GXG	1973.80	SKIP	
14	AXC	CXG	1983.92	YES	GTR7C
15	AXC	TXG	2043.25	SKIP	
16	CXA	GXC	2047.72	SKIP	
17	AXC	GXG	2211.16	SKIP	
18	CXG	TXG	2302.08	SKIP	
19	CXG	GXG	2341.46	SKIP	
20	CXG	GXC	2343.32	YES	GTR6C
21	AXC	AXT	2352.48	SKIP	
22	GXT	TXA	2363.72	YES	GTR5C
					
...	...	...	...	...	...

The graph-based reduction approach constructs the optimal model (GTR8C in Table 8) for the Ancestral Repeats dataset in 12 iterations (first column). The second and third column show which 2 contexts (or clusters) are proposed for merging; the fourth column shows the difference in log likelihood between the full 16-contexts model and the resulting 15-contexts model should only those 2 contexts given in the second and third column be merged; the fifth column shows the decision on the proposed merge (YES: the merge is performed; NO: the merge is not performed due to a lower cost alternative; SKIP: the merge is already present in the current clustering); the sixth column shows the resulting model when a merge operation is performed.

**Table 7:** Stepwise context reduction for the Ancestral Repeats dataset using the graph-based approach.

Model	Contexts	Annealing	Melting	log BF
GTR16C	16 (96)	[623.2; 638.2]	[645.5; 661.9]	642.2
				
GTR15C	15 (90)	[658.0; 672.1]	[665.0; 682.0]	669.2
GTR14C	14 (84)	[651.9; 668.4]	[664.3; 678.9]	665.9
GTR13C	13 (78)	[664.9; 679.6]	[676.4; 693.1]	678.5
GTR12C	12 (72)	[673.3; 689.1]	[685.3; 701.7]	687.4
GTR11C	11 (66)	[682.3; 697.9]	[693.5; 710.4]	696.0
GTR10C	10 (60)	[677.5; 693.4]	[697.5; 710.3]	694.7
GTR9C	9 (56)	[693.7; 707.6]	[710.4; 724.6]	709.1
**GTR8C**	**8 (48)**	**[699.3; 711.7]**	**[712.4; 727.5]**	**712.7**
GTR7C	7 (42)	[686.5; 700.0]	[705.1; 719.3]	702.7
GTR6C	6 (36)	[650.6; 663.0]	[651.2; 664.8]	657.4
GTR5C	5 (30)	[641.4; 652.3]	[639.2; 649.2]	645.5
				
GTR	1 (6)	-	-	0

The stepwise context reduction using our graph-based clustering approach reveals an optimal model with 8 clusters for the Ancestral Repeats dataset (GTR8C). It attains a log Bayes Factor of 712.7 (as compared to GTR1C), a significant improvement over the full context-dependent model (GTR16C) which has twice as many parameters. This model also outperforms the 10-clusters model determined by the likelihood-based clustering approach.

To illustrate step 4 of the algorithm (see the Materials & Methods section), we discuss the 5^th ^step of the graph-based reduction approach. The 4^th ^iteration has yielded a model allowing for twelve clusters: a first cluster consists of four contexts (CXC, GXC, GXT and TXC), a second cluster consists of two contexts (GXG and TXG) and ten other clusters consist of a single context. To calculate the current clustering cost, the cost of the branch connecting contexts GXG and TXG (1064.87 units) is added to the cost of all the interconnecting branches between the CXC, GXC, GXT and TXC contexts (see Table 6): CXC-GXC (1524.96), CXC-GXT (1925.79), CXC-TXC (872.10), GXC-GXT (1940.97), GXC-TXC (1777.24) and GXT-TXC (1300.93). The clustering in step 4 thus has a cost of 10,406.86 units. The proposed step in the 5^th ^iteration to expand the four-contexts cluster (CXC, GXC, GXT and TXC) with a fifth context, i.e. AXC, then results in a cost of 20,124.32 units. However, expanding the four-context cluster with CXA instead of AXC yields a cost of 19,423.12 units, as the CXA context lies reasonably close to all four contexts whereas AXC lies mainly close to the GXC context. Therefore, the four-context cluster is not expanded in this 5^th ^iteration.

**Figure 5:**
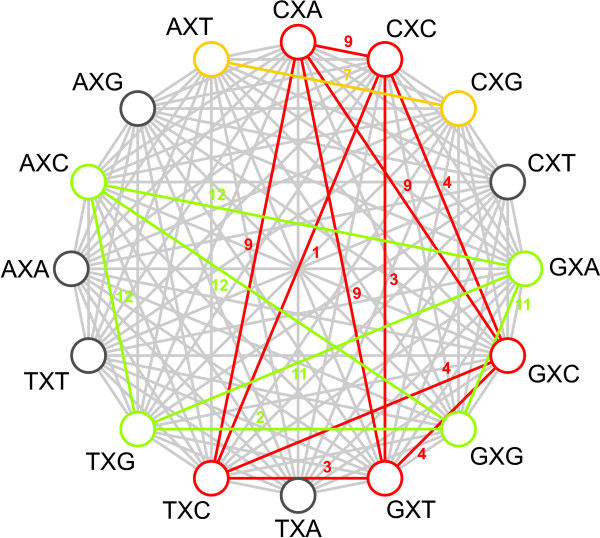
**Graphical representation of the graph-based context reduction approach**. The graphical representation of the graph-based reduction approach illustrates the coloring scheme to build the different clusters (the complete graph figure was obtained using GrInvIn; see [34]). The edges are labeled with the step of the graph-based algorithm during which they were colored. Given the large number of vertices in the graph, there are many possibilities of merging contexts into clusters. The coloring of nodes and vertices in this figure reveals an optimum of 8 clusters for the Ancestral Repeats dataset: 5 separate dark grey clusters (each containing a single context: AXA, AXG, CXT, TXA and TXT) and 3 colored clusters. The green cluster contains 4 contexts: AXC, GXA, GXG and TXG, the yellow cluster contains 2 contexts: AXT and CXG, and the red cluster contains 5 contexts: CXA, CXC, GXC, GXT, and TXC.

**Figure 6:**
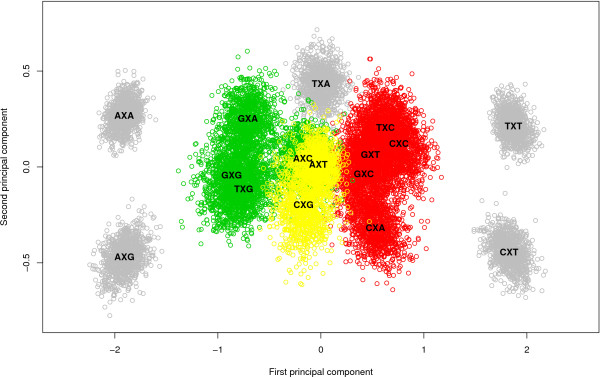
**Graphical representation of the graph-based optimal model for the Ancestral Repeats dataset**. The optimal model for the Ancestral Repeats dataset, using the graph-based clustering approach, reveals 8 clusters: 5 separate grey clusters (each containing a single context: AXA, AXG, CXT, TXA and TXT) and 3 colored clusters. The green cluster contains 4 contexts: AXC, GXA, GXG and TXG, the yellow cluster contains 2 contexts: AXT and CXG, and the red cluster contains 5 contexts: CXA, CXC, GXC, GXT, and TXC.

After further reductions, the graph-based method yields a different optimal model than the likelihood-based approach for the Ancestral Repeats dataset. The optimal clustering consists of 8 clusters (GTR8C; using a total of 48 parameters for the model) with a log Bayes Factor of 712.7 (see Table 7), thus yielding an improvement in model fit over the optimal clustering found by the likelihood-based approach. This model is illustrated by the dark grey bar in Figure 4. This model is reached in the 12^th ^step in Table 6, which corresponds to the graph coloring scheme shown in Figure 5. The 8 clusters are shown in Figure 6, which identifies 5 separate single-context clusters (for the evolutionary contexts AXA, AXG, CXT, TXA and TXT) and 3 clusters consisting of two or more contexts. A first cluster contains 2 contexts: AXT and CXG, a second cluster contains 4 contexts: AXC, GXA, GXG and TXG, and a final cluster contains 5 contexts: CXA, CXC, GXC, GXT, and TXC.

We have concluded earlier (see Table 4) that using more than four rate classes for the discrete approximation does not yield clear improvements in model fit. Hence, we have combined the optimal model, obtained with the graph-based reduction approach, with the assumption of varying rates across sites using four rate classes. We have compared this reduced model with varying rates across sites to the full context-dependent model with varying rates across sites (also with four rate classes). The full context-dependent model yields a log Bayes Factor of 960.1 (annealing: [935.5; 959.4], melting: [690.9; 984.6]), thereby clearly outperforming the full context-dependent model with equal rates (with a log Bayes Factor of 642.2) and the independent model with varying rates across sites (with a log Bayes Factor of 355.6). Further, the optimal model yields an even higher log Bayes Factor of 1029.8 (annealing: [1001.8; 1022.4], melting: [1037.0; 1058.1]), thereby conserving the increase in model fit obtained with equal rates (see Table 7).

#### Interpretation of the optimal model

The graph-based reduction approach yields the best performing context-dependent model for the Ancestral Repeats dataset, but the interpretation of the clustering of neighboring base compositions is far from obvious. To gain insight, we have studied the parameter estimates of the GTR model for the 16 neighboring base compositions, which have been reported and discussed in previous work (Baele et al., 2008). In a first step, we try to determine why the five contexts AXA, AXG, CXT, TXA and TXT are clustered separately in the 'optimal' context-dependent model. The AXA and AXG contexts have much higher rCT parameter estimates than all other contexts. For the AXG context, this could be attributed to a CpG effect, conditional on the preceding adenine. This might mean that, for the AXA context, a non-CpG methylation process is present although we are unaware of earlier reports of such a process in the AXA context. In previous work (see [12]), we already elaborated on the possibility of a TpA effect, especially in the AXA context. Such an effect could occur conditional on the preceding base, as is the case for the CpG effect.

Non-CpG methylation is the subject of many discussions in mammalian evolution. Woodcock et al. [29] reported that 55% of all methylation in human spleen DNA could be at dinucleotides other than CpG. In their analysis of mammalian DNA, Ramsahoye et al. [30] found that embryonic stem cells, but not somatic tissues, have significant cytosine-5 methylation at CpA and, to a lesser extent, at CpT. However, high relative rates between C and T have been observed in aligned gene/pseudogene sequences of human DNA in the past [31]. The reason for the separate clustering of the CXT and TXT contexts seems to be the lower than average (and again, than all other contexts) rCT parameter estimates (or the higher than average rAG parameter estimates). When considering that the CXT and TXT contexts can be found on the complementary strand of the AXG and AXA contexts, this makes perfect sense. This complementarity aspect is actually dominantly present in Figure 6 when considering the green and red clusters. Indeed, the green cluster contains contexts AXC, GXA, GXG and TXG while the red cluster contains all the complementary contexts, resp. GXT, TXC, CXC and CXA, further augmented with GXC. This latter context, along with the other symmetrical contexts (i.e. whose complementary context is the context itself) AXT, CXG and TXA correspond to a zero first principal component in Figure 6. This first principal component has a loading of 0.73 for the rAG parameter and -0.68 for the rCT parameter, with loadings for the other parameters all below 0.03. Hence, this principal component roughly measures the differences between the rAG and rCT parameter estimates. This explains why most of the clustering patterns in Figures 3 and 6 are retrieved in the rAG and rCT parameter estimates.

Only the separate TXA context cannot be explained using the transition estimates. Because both the rAG and rCT parameter estimates for this context are lower than average, the transversion estimates must be studied (see [12], online supplementary material). The TXA context has the highest rAT and rGT parameter estimates of all 16 contexts and the rAC parameter estimates are also above average, which seems to lead to a significantly differing evolutionary behavior when compared to all other contexts. This observation reinforces our opinion that modeling different substitution behavior of the transition parameters (as is mainly the case when modeling CpG effects) cannot by far account for the complexity of mammalian DNA evolution. Indeed, the separate clustering of the TXA context suggests that modeling different substitution behavior of the transversion parameters depending on the nearest neighbors can increase model fit. This is supported by a clear preference of the six-cluster model (GTR6C in Table 7), clustering TXA separately, over the five-cluster model (GTR5C in Table 7), which includes TXA in a large cluster with 11 other contexts.

We have already shown that modeling CpG effects, both dependent and independent of the preceding base, does not even come close to modeling a full context-dependence scheme based on the flanking bases in terms of model fit. The evolutionary patterns of sites with guanine as the 3' neighbor can nonetheless be seen to lie close in the principal components plot (see Figure 6). All four occurrences lie in the lower left section of the plot, even though only the GXG and TXG contexts are effectively clustered together. This reinforces our finding that CpG effects are only one aspect of context-dependent evolution and that CpG effects are dependent of the preceding base, with adenine being the most influential 5' neighbor resulting in a separate cluster. In terms of the transition parameter estimates, the CXG context has lower (higher) rCT (rAG) parameter estimates than both GXG and TXG, which explains why only GXG and TXG are clustered together. Apart from a small difference in the rCG parameter estimates, both contexts are very similar in their parameter estimates. The same goes for the AXT and CXG contexts, which only differ in the rCG parameter estimates. However, the cluster containing both GXG and TXG also contains the GXA and AXC contexts, meaning that this cluster (like all clusters determined) does not contain all the contexts with either identical 5' or 3' neighboring bases, i.e. the cluster containing GXG and TXG does not contain the AXG and CXG contexts. The GXA context differs from the GXG and TXG contexts in its parameter estimates for rCT and rAC, and a small difference for rAT. The transversion estimates of the AXC context yield no drastically differing observations when compared to those of the other three contexts in the cluster. The difference seems to lie in the transition parameters, where the AXC context is observed to have decreased rCT parameter estimates compared to the other contexts in the cluster.

One cluster left for discussion is the one containing the CXA, CXC, GXC, GXT and TXC contexts. Different from those sites with guanine as the 3' neighbor, those sites with cytosine as the 3' neighbor are clustered closer to one another, with CXC, GXC and TXC being part of the same cluster and thus only AXC being part of another cluster. In other words, as is the case when the 3' neighbor is guanine, those sites with adenine as their 5' neighbor are positioned away from the other occurrences with the same 3' neighbor. Apart from the rCG parameter estimates, the different contexts show only small differences in the parameter estimates. The CXA context has lower rCG parameter estimates than the other contexts in the cluster, which might explain why CXA is the last context to be added to the cluster in the graph-based reduction approach.

### The Nuclear SSU rRNA dataset

#### The likelihood-based reduction approach: results

Given the larger increase in model fit brought about by the graph-based reduction approach for the Ancestral Repeats dataset, we have opted to test this method on a previously analyzed nuclear small subunit ribosomal RNA dataset [12]. As this dataset is much smaller than the Ancestral Repeats dataset, calculation of the necessary log Bayes Factors is much faster and does not require applying our split calculation approach for the thermodynamic integration method. Instead, we have used a larger number of chain updates (*Q *= 1000) while increasing or decreasing *β *by 0.001 throughout the whole integration interval.

The starting point of the analysis of the nuclear SSU rRNA dataset is different from that of the Ancestral Repeats dataset in that the standard context-dependent model yields a log Bayes Factor of -17.65 compared to the independent GTR model (see Table 8), suggesting a large decrease in terms of model fit of the context-dependent model. While this could mean that there are no dependencies in this dataset, it might also be the result of overparameterization.

**Table 8:** Stepwise context reduction for the nuclear SSU rRNA dataset using the likelihood-based approach.

Model	Contexts	Annealing	Melting	log BF
GTR16C	16 (96)	[-21.25; -15.74]	[-19.29; -14.31]	-17.65
				
GTR15C	15 (90)	[-17.17; -13.45]	[-14.19; -10.30]	-13.78
GTR14C	14 (84)	[-14.05; -10.21]	[-10.09; -5.13]	-9.87
GTR13C	13 (78)	[-11.07; -7.85]	[-6.80; -3.42]	-7.28
GTR12C	12 (72)	[-2.61; 1.25]	[-0.71; 3.31]	0.31
GTR11C	11 (66)	[-0.11; 3.55]	[0.62; 3.77]	1.96
GTR10C	10 (60)	[-2.33; 1.28]	[8.76; 13.06]	5.19
GTR9C	9 (54)	[6.94; 10.27]	[9.03; 13.94]	10.05
GTR8C	8 (48)	[9.54; 12.82]	[12.12; 16.21]	12.67
GTR7C	7 (42)	[12.80; 16.45]	[18.26; 22.96]	17.62
**GTR6C**	**6 (36)**	**[13.75; 16.89]**	**[21.71; 26.13]**	**19.62**
GTR5C	5 (30)	[15.87; 18.89]	[17.86; 22.19]	18.70
GTR4C	4 (24)	[13.03; 16.88]	[17.38; 20.71]	17.00
GTR3C	3 (18)	[9.43; 12.63]	[12.61; 15.66]	12.58
GTR2C	2 (12)	[11.69; 15.19]	[12.84; 16.58]	14.08
				
GTR	1 (6)	-	-	0

The stepwise context reduction using the likelihood-based clustering approach reveals an optimal model with six clusters for the nuclear SSU rRNA dataset (GTR6C). It attains a log Bayes Factor of 19.62 (as compared to GTR1C), a significant improvement over the full context-dependent model (GTR16C).

**Figure 7:**
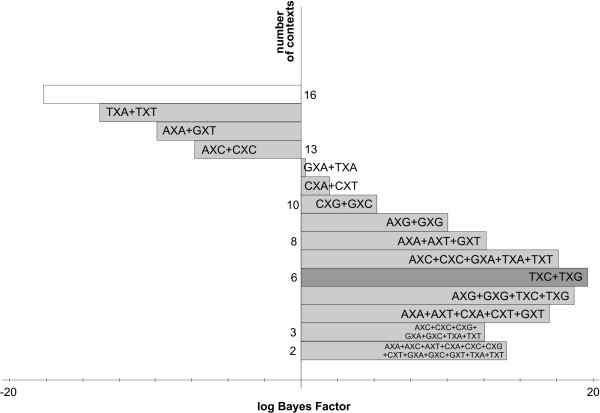
**Stepwise graph-based clustering of the nuclear SSU rRNA data**. The stepwise clustering of contexts using the likelihood-based clustering approach shows, from top to bottom, the subsequent merges of contexts for the nuclear SSU rRNA dataset. The starting point is a full (96-parameter) context-dependent model, shown in white. The optimal model has 6 clusters and 36 parameters.

**Figure 8:**
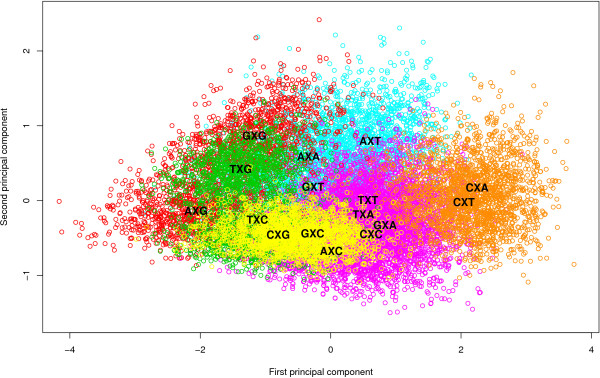
**Graphical representation of the likelihood-based optimal model for the nuclear SSU rRNA dataset**. The optimal model for the nuclear SSU rRNA dataset, as obtained by both the likelihood-based and the extended clustering approach, has 6 clusters: the red cluster contains 2 contexts: AXG and GXG, the green clusters contains 2 contexts: TXC and TXG, the light blue cluster contains 3 contexts: AXA, AXT and GXT, the yellow cluster contains 2 contexts: CXG and GXC, the purple cluster contains 5 contexts: AXC, CXC, GXA, TXA and TXT, and the orange cluster contains 2 contexts: CXA and CXT.

The first four reductions made by the likelihood-based reduction approach yield a context-dependent model with equal model fit to that of the independent GTR model. Further reductions yield a context-dependent model consisting of six contexts, which significantly outperforms the independent model with a log Bayes Factor of 19.62. This indicates that the true context-dependent effects were initially undetectable due to the drastic increase in parameters. As we show here, a careful model-building strategy can unveil the important evolutionary contexts, leading to an increased performance in terms of model fit. This will become an even more important aspect when modelling additional dependencies. The stepwise clustering of contexts for the likelihood-based clustering, in terms of the log Bayes Factor, can be seen in Figure 7 and Table 8. The optimal clustering using this likelihood-based reduction approach can be seen in Figure 8.

#### The extended likelihood-based reduction approach: results

Because the nuclear SSU rRNA dataset is relatively small, it is feasible to re-estimate the evolutionary parameters after each merge of contexts or clusters. This allows for a more accurate calculation of the posterior variances for each cluster of contexts and may result in a different context-dependent model. We call this approach the extended likelihood-based reduction approach and compare its results to the regular likelihood-based reduction approach. Note that re-estimating a posterior variance would take over four days of computation time in the Ancestral Repeats dataset, which in turn would lead to over sixty days of computation time in total until all Bayes Factor calculations can be started.

As can be seen from Table 9, the extended likelihood-based reduction approach yields an identical optimal model as the simple likelihood-based reduction approach although the path that both approaches take towards this optimal model is different (data not shown). This illustrates that the simple approach may yield a good approximation and that it is not always necessary to perform tedious calculations to achieve a decent parameter-performance trade-off. The approximation may become poorer, however, as the clustered contexts are further apart (because this increases the difference between empirical and posterior variance of each cluster).

**Table 9:** Stepwise context reduction for the nuclear SSU rRNA dataset using the extended likelihood-based approach.

Model	Contexts	Annealing	Melting	log BF
GTR16C	16 (96)	[-21.25; -15.74]	[-19.29; -14.31]	-17.65
				
GTR15C	15 (90)	[-17.17; -13.45]	[-14.19; -10.30]	-13.78
GTR14C	14 (84)	[-14.05; -10.21]	[-10.09; -5.13]	-9.87
GTR13C	13 (78)	[-9.48; -6.19]	[-7.70; -3.72]	-6.77
GTR12C	12 (72)	[-6.99; -3.70]	[-3.84; -0.05]	-3.62
GTR11C	11 (66)	[-0.60; 2.92]	[2.97; 7.62]	3.23
GTR10C	10 (60)	[4.85; 8.77]	[6.62; 11.95]	8.05
GTR9C	9 (54)	[9.05; 12.46]	[8.55; 13.02]	10.77
GTR8C	8 (48)	[8.07; 11.26]	[8.68; 12.44]	10.11
GTR7C	7 (42)	[15.82; 19.60]	[15.09; 21.19]	17.93
**GTR6C**	**6 (36)**	**[13.75; 16.89]**	**[21.71; 26.13]**	**19.62**
GTR5C	5 (30)	[15.87; 18.89]	[17.86; 22.19]	18.70
GTR4C	4 (24)	[14.10; 17.69]	[14.30; 18.90]	16.25
GTR3C	3 (18)	[11.20; 14.99]	[15.22; 18.24]	14.91
GTR2C	2 (12)	[8.37; 11.57]	[13.14; 17.04]	12.53
				
GTR	1 (6)	-	-	0

The stepwise context reduction using the extended likelihood-based clustering approach reveals the same optimal model with six clusters as the simple likelihood-based clustering approach for the nuclear SSU rRNA dataset.

#### The graph-based reduction approach: results

**Figure 9:**
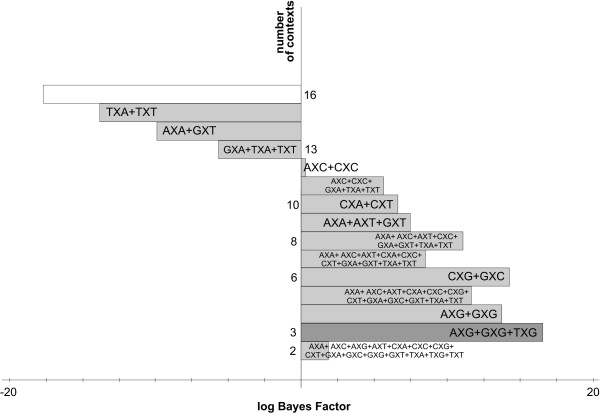
**Stepwise graph-based clustering of the nuclear SSU rRNA data**. The stepwise clustering of contexts using the graph-based clustering approach shows, from top to bottom, the subsequent merges of contexts for the nuclear SSU rRNA dataset. The starting point is a full (96-parameter) context-dependent model, shown in white. The optimal model has 3 clusters and 18 parameters.

**Figure 10:**
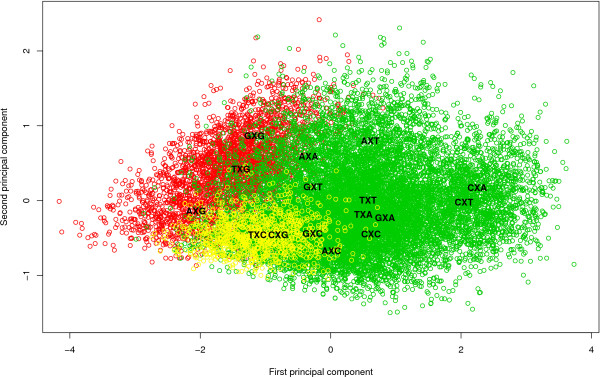
**Graphical representation of the graph-based optimal model for the nuclear SSU rRNA dataset**. The optimal model for the nuclear SSU rRNA dataset, using the graph-based clustering approach, reveals only 3 clusters. The yellow cluster contains one single context: TXC, the red cluster contains 3 contexts: AXG, GXG and TXG and the green cluster contains all 12 remaining contexts.

In this dataset, the graph-based reduction approach yields an optimal model with three clusters, containing only 18 parameters. The stepwise reduction, starting from the full context-dependent model, can be seen in Figure 9, with the corresponding log Bayes Factors for each step given in Table 10. A representation of the optimal clustering scenario is shown in Figure 10, where the three clusters can be identified: a first (red) cluster containing the contexts AXG, GXG and TXG, a second (yellow) cluster containing the single context TXC and a large (green) cluster containing all remaining 12 contexts. The log Bayes Factor for this model equals 16.55 when compared to the independent model, which is just below the log Bayes Factor generated by the optimal model using the likelihood-based reduction approach, although the two model performances are not significantly differing from one another. Note however that the confidence intervals in both directions seem to overlap more using the graph-based reduction approach, resulting in higher accuracy for the calculated log Bayes Factors.

**Table 10:** Stepwise context reduction for the nuclear SSU rRNA dataset using the graph-based approach.

Model	Contexts	Annealing	Melting	log BF
GTR16C	16 (96)	[-21.25; -15.74]	[-19.29; -14.31]	-17.65
				
GTR15C	15 (90)	[-17.17; -13.45]	[-14.19; -10.30]	-13.78
GTR14C	14 (84)	[-14.05; -10.21]	[-10.09; -5.13]	-9.87
GTR13C	13 (78)	[-7.29; -4.07]	[-8.00; -3.25]	-5.65
GTR12C	12 (72)	[-2.61; 1.25]	[-0.71; 3.31]	0.31
GTR11C	11 (66)	[1.75; 5.55]	[6.05; 9.25]	5.65
GTR10C	10 (60)	[3.50; 7.11]	[5.53; 10.37]	6.63
GTR9C	9 (54)	[2.79; 6.32]	[8.24; 12.51]	7.47
GTR8C	8 (48)	[8.43; 12.07]	[10.37; 13.48]	11.09
GTR7C	7 (42)	[4.21; 7.25]	[9.53; 13.04]	8.51
GTR6C	6 (36)	[11.71; 14.99]	[12.83; 17.55]	14.27
GTR5C	5 (30)	[8.97; 12.70]	[10.10; 14.86]	11.66
GTR4C	4 (24)	[11.44; 15.02]	[12.18; 16.35]	13.75
**GTR3C**	**3 (18)**	**[15.30; 17.92]**	**[14.86; 18.11]**	**16.55**
GTR2C	2 (12)	[-1.61; 2.74]	[1.04; 5.31]	1.87
				
GTR	1 (6)	-	-	0

The stepwise context reduction using the graph-based clustering approach reveals an optimal model with 3 clusters for the nuclear SSU rRNA dataset (GTR3C). It attains a log Bayes Factor of 16.55 (as compared to GTR1C), a significant improvement over the full context-dependent model (GTR16C).

#### Interpretation of the optimal model

The optimal model for the nuclear SSU rRNA dataset consists of six clusters and has 36 parameters. The reasons for this specific clustering scenario can be identified by considering the parameter estimates for the 96 parameters of the model, as reported in earlier work [12]. The fact that there is support for the presence of CpG effects in this dataset only leads to the clustering of contexts AXG and GXG while TXG is clustered with TXC and CXG with GXC. The TXG context has higher estimates for the rGT parameter and its substitution behavior bears more resemblance to that of TXC. The CXG context has much higher rAG estimates compared to the AXG and GXG contexts, with the highest rAG estimates for the CXA and CXT contexts which are clustered together. The larger clusters are harder to explain as there are more differences between the individual parameters. For example, it can be seen that the largest cluster has more or less average estimates for all six parameters, although this is not the case for the rAT parameter where the contexts with higher estimates are clustered together with the exception of the TXC context.

Note that the increases in terms of model fit of these context-dependencies (as expressed through the log Bayes Factor) might seem less relevant in this dataset given the drastic increase brought about by assuming varying rates across sites, i.e. a log Bayes Factor of 499.10 units compared to the independent GTR model (see [12]). In other datasets (e.g. the Ancestral Repeats dataset), however, the opposite is true. Hence, a context-dependent model might also be useful for those datasets where no drastic increases in terms of model fit are brought about by assuming varying rates across sites, such as the frequently analyzed dataset of six ψη-globin pseudogenes [32] which originally appeared in the work of Miyamoto et al. [33].

## Conclusion

In this work, we have introduced a parallelization of the model-switch integration method, as proposed by Lartillot and Philippe [18], to accurately calculate Bayes Factors for model comparisons on large datasets. The main advantage of our approach consists of the ability to split the calculations of the Bayes Factors over different computers, to minimize the time to yield results at the cost of only a slightly larger variation around the actual estimates. Given the increase in computation power using cluster computing during recent years, the ability to split certain calculations over different computers is a valuable aid in analyzing large datasets, such as the one studied in this work. Using thermodynamic integration with bidirectional checks, we have evaluated 28 models against the independent GTR model for the Ancestral Repeats dataset, a large dataset consisting of 10 sequences each containing 114,726 sites. As indicated by Lartillot and Philippe [18], thermodynamic integration requires tremendous amounts of CPU time.

Calculations for all the log Bayes Factors for the Ancestral Repeats dataset reported in this work were split over 20 processors, using the settings shown in Tables 2 and 3. Calculating one of these 20 integration intervals takes about 5 days on an Intel Xeon 2.4 Ghz processor. Given that bidirectional checks were performed and that 28 models were evaluated, this amounts to over 15 years of computation time that was used to obtain the log Bayes Factors and the corresponding confidence intervals for the Ancestral Repeats dataset. Given the magnitude of the improvements shown by our models, we did not have to use the most stringent settings for the thermodynamic integration method, which would obviously have increased computational demands even more.

Using this split-calculation approach, we have shown that significant improvements in terms of model fit (calculated through the use of Bayes Factors) of context-dependent models are possible, by optimizing the performance-parameter tradeoff. Indeed, standard context-dependent models tend to estimate too many parameters, reducing the amount of data present to estimate each pattern accurately. Furthermore, many parameters in such context-dependent models might be nearly equal to one another, meaning that several parameters in the model may just as well be replaced by one single parameter.

Using both our likelihood- and graph-based clustering approaches, we have shown that evolutionary patterns in the Ancestral Repeats dataset do not solely depend upon a similar identity of either the 5' or 3' neighboring base. We have shown that even CpG effects depend upon the 5' neighbor of the site under consideration and that many more substitution patterns are present in the data, as the CpG effect yields a much smaller increase in model fit than the full context-dependent model. The clustering results of our optimal context-dependent model, obtained through our graph-based reduction approach, confirm this finding. No cluster contains all four occurrences of the neighboring base combinations, conditional on either 5' or 3' neighboring base. At most, three contexts with either a similar 5' or 3' neighboring base are clustered together, i.e. CXC, GXC and TXC appear in a cluster together with CXA and GXT.

In contrast with the calculation for the Ancestral Repeats dataset, each model comparison for the nuclear SSU rRNA dataset could be performed in a single run of 5 days. Using the likelihood- and graph-based reduction approaches, we calculated all 15 reduction steps from the full context-dependent model to the independent model and found that the optimal clustering scheme contains six clusters. The likelihood-based approach yields the largest improvement in terms of model fit for this nuclear SSU rRNA dataset and also overthrows the initial conclusion that a full context-dependent model does not explain the data better than an independent model. This clearly illustrates that great care must be taken when building complex models that contain many parameters. A similar conclusion can be drawn for other context-dependent approaches (see e.g. [13,14]) than the one used in this paper [12].

One might argue that the visualization of the different contexts on a scatter plot (see Figures 3, 6, 8 and 10) along with the credibility intervals for all the available parameters of the context-dependent model (see [12]) provide enough clues to determine an optimal clustering scenario. While certain aspects of the optimal clustering scenario may be retrieved through the use of visualization aids, many other aspects of the clustering would then depend upon subjective decisions, thereby reducing reproducibility. To avoid the need for subjective decisions and restriction to the first two principal components, we have presented two automatic approaches which we found to yield better context-dependent models in a straightforward manner.

The approaches discussed in this work can be used with different underlying evolutionary models. While we have assumed context-dependent evolution using 16 different GTR models in this work, we have assumed the equilibrium frequencies to be context-independent. In other words, we assume a stationary equilibrium distribution for the base frequencies. Such an assumption may be overly restrictive and its relaxation in a context-dependent framework may result in a larger increase in terms of model fit at the expense of increased computation times.

The computational complexity of the model selection approaches presented in this paper may be reduced by using a reversible jump MCMC (RJMCMC) approach [35]. By designing appropriate trans-dimensional jumps, flexible increases and decreases of the number of evolutionary contexts can be implemented. Analyzing the posterior distribution of the identities of the models that are required can then identify the optimal combination of contexts to be used in subsequent analyses.

Finally, the approaches discussed in this work can be applied to a wider range of problems whereby decisions on the equality of parameter values must be taken. For instance, when assuming branch- or lineage-specific evolutionary models (using the independence assumption), the number of models may initially be too large as not each branch or lineage may require a specific model differing from all other models present in the analysis.

## Authors' contributions

GB initiated the study, co-designed the split calculation approach, designed the graph-based reduction approach, performed all the analyses, programmed the software routines and wrote a first complete version of the manuscript. YVdP contributed biological expertise to the analyses and edited the manuscript. SV contributed statistical expertise to the analyses, co-designed the split calculation approach, designed the likelihood-based reduction strategy and edited the manuscript. All authors read and approved the final manuscript.
